# A view to kill

**DOI:** 10.1186/1741-7007-10-18

**Published:** 2012-03-05

**Authors:** Thomas W Holstein

**Affiliations:** 1 Centre for Organismal Studies (COS) Heidelberg, Heidelberg University, INF 230, 69120 Heidelberg, Germany

## Abstract

Genome and proteome data from *Hydra magnipapillata *have opened the way for the molecular analysis of an ancient nervous system, which includes stinging cells, an unusual neurosensory and neurosecretory cell type. They hold some surprises for the mechanisms and evolution of sensory transduction that could not have been anticipated from what has been learned from flies and vertebrates. Research in *BMC Biology *now implicates the ancient opsin-mediated transduction pathway in the neuronal control of stinging cell discharge.

See research article http://www.biomedcentral.com/1741-7007/10/17

## Commentary

For swimmers close to Pacific coastlines, a slight touch by the tentacles of a box jellyfish can be dangerous or even fatal. One of the most sophisticated cellular inventions in animal evolution, the cnidarian stinging cell is responsible for this toxicity. These cells (also named cnidocytes or nematocytes) are characteristic of cnidarians, a group of animals that include jellyfish, sea anemomes and freshwater polyps such as *Hydra*. Cnidarians diverged from bilaterians early in metazoan evolution, and the study of their stinging cells and its neuronal control brings insights of relevance to the evolutionary origins of sensory perception.

## Discharge of stinging cells and its control

Each nematocyte contains one giant vesicle that is derived from the Golgi apparatus, which is packed with neurotoxic and hemolytic venoms and a coiled spiny tubule inside a capsule, the nematocyst (cnidocyst, cnida) [1]. The 'sting' of a jellyfish shares more similarity with fast neurotransmitter release at a synapse than with the sting of a wasp or stinging nettle, because a nematocyst vesicle unloads its contents (that is, the capsule) by ultra-fast exocytosis [2]. This process is initiated when a prey deflects the ciliary mechanoreceptor, triggering an action potential and the opening of calcium-channels [1]. During the discharge of hydra stenoteles, one of the most elaborated nematocyst types, the barbed part of the tubule is accelerated in less than 700 ns, generating accelerations greater than 5,000,000 *g *and a pressure of up to 7 GPa, sufficient to penetrate even the cuticles of crustaceans [2]. This ultrafast process is powered by a high osmotic pressure of 150 bars that elastically stretches the capsule wall to which the long and barbed nematocyst tubule is attached. A proteome analysis of the secretome of *Hydra magnipapillata *[3] provides molecular clues as to how the elasticity and tensile strength of the capsule wall is achieved, featuring unique structural proteins with elastic properties. Minicollagens constitute a major subgroup of these nematocyst-specific extracellular proteins, but another important constituent, dubbed 'cnidoin' is non-collagenous, resembling the spider silk protein spidroin-2.

**Figure 1:**
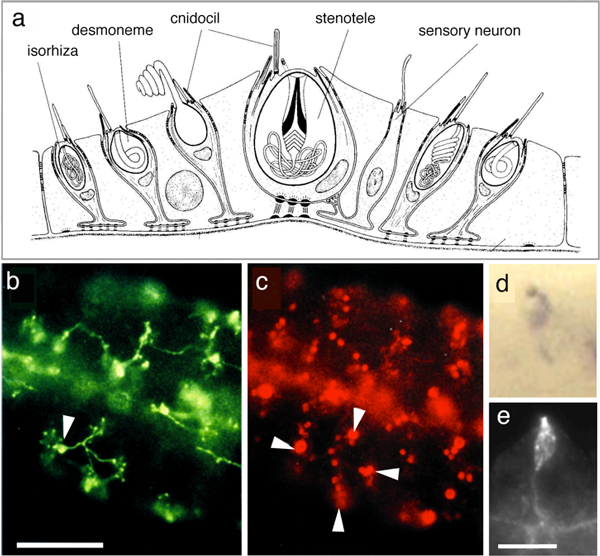
**Cellular organization of a battery cell complex in *Hydra***. **(a) **Schematic representation showing the location of the different cell types in a battery cell complex. (Adapted from Figure 1e of [6]). **(b, c) **Double staining of tentacle tissue of *Hydra magnipapillata *with the monoclonal antibodies (b) NVl (neuron-specific, green) and (c) H22 (nematocyte-specific, red) shows innervation of the nematocytes (arrowheads indicate (b) the NV1+ nerve cell body and (c) four large nematocysts in different battery cells). (Reproduced from Figure 1a, b of [6]). **(d, e) **Staining of tentacle tissue with (d) NV1 monoclonal antibodies and (e) the CnAsh probe, which detects the product of *achaete-scute*, a gene specifically expressed in nematocytes and the sensory neurons of battery cells. (Reproduced from Figure 5 of [8]). Scale bar: 25 μm (b, c); 5 μm (e, d).

Much remains to be learned about the control of nematocyst discharge. In all cnidarians, discharge can be modulated by chemo- and mechanoreceptors, and it is also under inhibitory control upon satiation [4,5]. In sea anemones, the regulation of nematocyte exocytosis involves an adjacent mechanoreceptor complex, which consists of a sensory neuron with a kinocilium, surrounded by a bundle of stereocilia arising from hair cells [4]. The chemoreceptors are probably G-protein coupled and mechanotransduction involves transient receptor potential (TRP) ion channels, which are responsible for detecting a range of chemical and mechanical stimuli in vertebrates. Of particular interest is TRPA1, a pain and mechanoreceptor in vertebrates [5]. This stress sensor also occurs in the hair bundles of sensory neurons that are associated with nematocytes in sea anemones [4]. TRPA1 is encoded by the genome of *Hydra magnipapillata*, but we do not yet know whether it is also involved in the control of nematocyst discharge in *Hydra*. The hair-bundle-like sensory apparatus of *Hydra *is formed by the nematocyte itself, and it surrounds the kinocilium (known as the cnidocil) and the docking site of the nematocyst vesicle (Figure 1a). Also in *Hydra*, a sensory cell bearing a single cilium can be found in close proximity to the nematocytes [6]. This sensory cell was proposed to be a chemoreceptor, and it innervates up to 30 different nematocytes [6]. The latter are arranged in battery complexes (Figure 1) and as many as three batteries of nematocytes can be connected in this way (Figure 1).

## Opsin-mediated phototransduction modulates nematocyte discharge

The work of Plachetzki and colleagues [7] now shows that the sensory neuron of the battery complex is a photoreceptor that controls the discharge process. These authors have demonstrated that this sensory neuron co-expresses components of the phototransduction cascade: opsin, a cyclic nucleotide gated (CNG) ion channel, and arrestin. After carrying out behavioral trials with *Hydra*, Plachetzki and colleagues concluded that different light intensities elicit significant effects on cnidocyte discharge. The readiness of stenoteles to discharge was significantly smaller under bright light conditions than in dim light. Treatment of *Hydra *with an inhibitor of CNG ion channels (cis-diltiazem) rescued this inhibition.

Photosensitive behavior of *Hydra *has been observed previously, but this is the first study to provide clear evidence that it can be traced to a cellular receptor. The surprising new findings are that sensory neurons in the battery-cell complex of *Hydra *tentacles exhibit this photosensitivity and, more importantly, that light information is used to control nematocyte discharge. The authors present several hypotheses that might explain light-regulated nematocyte discharge. Of these hypotheses, one is particularly intriguing as it assumes a further optimization of discharge: a shadow being cast by the prey on a battery complex could enhance the likelihood that stenoteles hit their target [7].

Nematocytes are the major cell population in a *Hydra*, and they are maintained by a stem cell system that also gives rise to nerve cells. It is therefore parsimonious to assume that the observed integration of light information by opsin-based signaling into the control of nematocyte discharge is under strong selective pressure. This selective pressure might even have led to the formation of ocelli in hydrozoan medusae (for example, in *Podocoryne carnea*) or even to the complex lens eyes in box jelly fishes (for example, in *Carybdea marsupialis *or *Tripedalia cystophora*).

**Figure 2:**
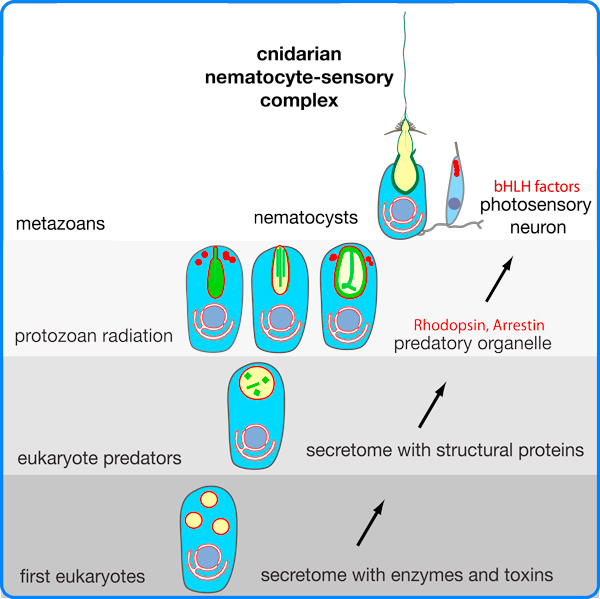
**Schematic representation of the hypothetical evolution of the cnidarian nematocyst**. The secretome of early eukaryotes exhibited secretory vesicles that contained enzymes used for extracellular digestion. It evolved structural proteins with biomechanical properties that finally gave rise to a variety of protozoan extrusive organelles, which exhibit sophisticated discharge mechanisms. Some of these organelles are structurally similar to nematocysts, suggesting that nematocysts might have evolved from the extrusomes of a non-cnidarian precursor. Note that core components of the phototransduction cascade evolved in protozoans [9], as indicated by red vesicles and lettering. A nematocyte-sensory receptor complex evolved only in multicellular cnidarians/metazoans (Adapted from Figure 6 of [3]).

During development, the formation of vertebrate light- and mechanosensors (eyes and ears) is tightly coupled. In vertebrates, the transcription factor *Pax6 *controls eye and *Pax2 *ear formation. In addition, the basic helix-loop-helix (bHLH) gene *achaete-scute *is co-utilized in hair and retina cell differentiation [7]. The identification of an ancestral *Pax2/6 *gene in *Hydra *and *Nematostella*, and the specific expression of *achaete-scute *in nematocytes and in the sensory neuron of battery cells [6,8] (Figure 2), suggest that the evolution of two major modes of sensation has a strong molecular link to the regulation of nematocyte discharge.

## Origin of photic and mechanic sensation - linked to cnidae?

So how did phototransduction evolve? Plachetzki and colleagues propose that the regulation of nematocyte discharge by opsin-mediated phototransduction predated this pathway's function in cnidarian eyes. This is a parsimonious statement, but despite the fact that opsins and nematocytes represent the most ancient characters in cnidarian evolution, one could go one step further. Opsins had already evolved in protozoans, and the phototactic behavior of some heterotrophic dinoflagellates is clearly based on sensory rhodopsins [9]. Notably, nematocyst-like organelles have even been described in dinoflagellates and other protozoans, a fact that argues for an evolutionary origin of nematocysts that predates multicellularity [2,3].

This opens the attractive possibility that there was a eukaryotic cell at the base of metazoan evolution that had an extrusive organelle and the molecular repertoire for photic and mechanic sensation. If descended from a protozoan ancestor of early metazoans, such cells might have arisen by lateral gene transfer (or organelle transfer) from a protist. Alternatively, the first multicellular animals directly derived from protozoans exhibiting extrusive organelles and signal transduction cascades for photic and stress perception. Although none of the currently sequenced protist genomes contains a homolog of the nematocyst-specific proteins with biophysical properties that might account for the high speed discharge of such organelles [3], this situation could change in the future.
